# The mechanism of deceleration of nucleation and crystal growth by the small addition of transition metals to lithium disilicate glasses

**DOI:** 10.1038/srep25451

**Published:** 2016-05-06

**Authors:** Katrin Thieme, Isak Avramov, Christian Rüssel

**Affiliations:** 1 Otto-Schott-Institut für Materialforschung, Jena University, Fraunhoferstr. 6, 07743 Jena, Germany; 2 Institute of Physical Chemistry, Bulgarian Academy of Science, Sofia, Bulgaria

## Abstract

The addition of small amounts of niobium or tantalum oxide to lithium disilicate glass provokes a drastic decrease of the steady-state nucleation rates and the crystal growth velocities. The viscosity of the residual glassy matrix is considered as a function of the crystallization degree in the course of a non-isothermal crystallization. For simplification, a homogeneous distribution of the added oxides in the glass matrix is assumed. While the viscosity initially decreases, it significantly increases again for higher crystallization degrees hindering crystal growth. However, it was shown that the additives are enriched at the crystal interface. Several possible reasons for the inhibition of nucleation and growth kinetics such as viscosity, interfacial energy crystal/glassy phase, thermodynamic driving force or impingement rate are discussed. Since the crystallization front is blocked by the additives the impingement rate is decreased with increasing additive concentration. Since small concentrations of Nb_2_O_5_ and Ta_2_O_5_ have a drastic effect on the nucleation, these components should be enriched at the interface crystal/glass. This will only take place, if it leads to a decrease in the interfacial energy. Since this effect alone should result in an increase of the nucleation rate, it must be overcompensated by kinetic effects.

In the past decades, nucleation and crystal growth kinetics of glasses has been intensively studied, preferentially in isochemical systems where the crystalline phase possesses the same chemical composition as the parent glass. One of the model systems for homogeneous nucleation is stoichiometric lithium disilicate glass as detailed thermodynamic and kinetic data are available^1^,^2^,^3^,^4^,^5^,^6^. In isochemical systems, the composition of the glass remains constant during nucleation and the crystal growth velocity is time-independent. Strictly spoken, each glass always contains more or less high quantities of impurities, and hence a system cannot be ideally isochemical. The impurities are either incorporated into the crystal or are enriched at the crystallization front leading to the formation of concentration gradients which decelerate crystal growth. In the latter case, the chemical composition of the residual glassy matrix changes during the course of crystallization. It is also possible that the viscosity in a diffusion layer around the crystals increases resulting in the formation of a diffusion barrier, i.e. in the decrease of the diffusion coefficients which also decelerates crystal growth^7^,^8^,^9^,^10^,^11^,^12^. The occurrence of self-limited crystal growth in some glass systems was theoretically described^13^,^14^,^15^ and diffusion barriers surrounding growing crystals have been experimentally proven^16^,^17^,^18^,^19^,^20^. However, the viscosity at the interface can also be lowered by the occurrence of network modifiers leading to an increased mobility and hence to a higher crystal growth velocity^13^. It is also observed for Fe-Mn-O nanoparticles in a SiO_2_/Na_2_O/Fe_2_O_3_/MnO glass that the formation of a rigid shell around crystals does not impair the diffusion of the ions which is related to a decreased glass transition temperature of the shell region^21^. The effect of small amounts of oxides (e.g. ZrO_2_^22^,^23^, TiO_2_^24^,^25^) added to specific glass compositions in order to promote nucleation has intensively been investigated in the past. However, the contrary effect of nucleation inhibition has only been scarcely investigated. Recently, it was shown that 1 or 2 mol% Al_2_O_3_, La_2_O_3_, TiO_2_^26^, ZrO_2_^27^, Nb_2_O_5_ or Ta_2_O_5_^28^ may affect the nucleation and growth kinetics if added to stoichiometric lithium disilicate glass. Depending on the chosen additive and its concentration, the steady-state nucleation rates may be affected in various degrees. For example, adding 2 mol% of La_2_O_3_ or Nb_2_O_5_ may provoke a decrease in the nucleation rates by up to 3 orders of magnitude and an extreme increase of the induction times^26^,^28^. Moreover, the crystal growth velocities are drastically decreased.

Although the effect of nucleation inhibition has recently been experimentally described, the reasons for this effect are still unknown. Thus, we try to analyze whether several parameters such as viscosity, interfacial energy glass/crystal or the thermodynamic driving force may be considered as key factors for this effect. Moreover, the change in viscosity of the residual glass matrix is regarded as a function of the crystallization degree, for both isothermal and non-isothermal conditions.

## Experimental Section

### Glass preparation

Glasses with the compositions c M_2_O_5_ · (100-c)Li_2_Si_2_O_5_ (c =  0.1, 1 or 2 mol%, M =  Nb, Ta) were prepared from the raw materials SiO_2_ (Sipur A1, Bremthaler Quarzitwerke), Li_2_CO_3_ (Polskie Odczynniki Chemiczne Gliwice), Nb_2_O_5_ (Ventron) and Ta_2_O_5_ (Alfa Aesar). The glass batches (usually for 200 g glass) were melted in a platinum crucible in the temperature range from 1450 to 1510 °C in an inductive furnace for 0.5–1 h followed by a stirring process for 1.5 h using a frequency of 40–60 min^−1^. Afterwards the melts were soaked for further 15 min at the same temperature without stirring. In order to avoid crystallization during cooling two glass blocks were cast on a copper and brass block and subsequently transferred to a cooling furnace preheated to 475–495 °C. The furnace was immediately switched off allowing the glass to cool to room temperature and to release stresses. The prepared glasses were stored in a desiccator using P_2_O_5_ as drying agent.

### Differential thermal analyses

For the determination of the glass transition and crystallization temperatures differential thermal analyses were performed using a *SHIMADZU DTA 50* and a *Linseis DSC PT1600*. In order to minimize the effect of surface crystallization bulk samples were used. Here, 60 mg glass powder was remelted in a DTA platinum crucible above the liquidus temperature for 5 min and then cooled in air. The remelted samples were heated with 10 K/min to 1100 °C, kept for 15 min and cooled down with 10 K/min to room temperature. After the measurements baseline corrections were made.

For the determination of the activation energy for crystallization, DSC scans with different heating rates in the range 5–20 K/min were carried out. The activation energy of the overall crystallization process E was determined using the following equation^29^.


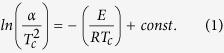


where α is the heating rate, T_c_ the peak crystallization temperature and R the gas constant. To estimate the melting enthalpies ∆ H_m_, DSC scans with a heating rate of 20 K/min up to 1100 °C were performed. Since ∆ H_m_ is proportional to the melting peak area, this value can be roughly determined using DSC^30^. In the case of the samples Nb1 and Nb2 two melting peaks occur due to surface and volume crystallization^28^. Possibly, there is a narrow fraction of very small crystals possessing a lower melting point. Therefore, the superimposed peaks were separated using the software Fityk and the areas of both peaks were determined and summated.

The glass transition temperatures T_g_ of partially crystallized samples were determined by dilatometry. Therefore, cylindrical samples previously crystallized at a certain temperature for different holding times (Nb1 and Ta1: nucleation at 489 °C for 2 h and growth at 640 °C; Nb2: nucleation at 489 °C 65 h and growth at 650 °C) were heated with 5 K/min in a *Netzsch DIL 420 PC*.

### Viscosity measurements

The viscosity measurements were performed using a rotation viscometer *Bähr VIS 403* (10^1.3^ to 10^3^ dPa · s, rotation speed 250 min^−1^, cooling rate 5 K/min) and with a beam bending viscometer *Bähr VIS 401* (10^9^ to 10^12.5^ dPa · s, heating rate 10 K/min, glass bars with the dimension 5 ×  5 ×  50 and 5 ×  4 ×  50 mm^3^). The measured viscosities were fitted using the Avramov-Milchev-equation,


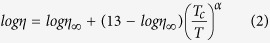


where the parameter η _∞_ describes the viscosity at T→ ∞ and α is the dimensionless fragility parameter. T_c_ characterizes the temperature at which log η  =  13 dPa ∙ s and is close to the experimentally measured glass transition temperature T_g_.

## Results

### Nucleation rates and crystal growth velocities of the glass Nb0.1

In a recently published paper^28^, 1 or 2 mol% Nb_2_O_5_ or Ta_2_O_5_ were added to stoichiometric lithium disilicate glass resulting in drastically decreased steady-state nucleation rates by of up to three orders of magnitude. In order to analyze the effect of trace impurities, a lithium disilicate glass was doped with 0.1 mol% Nb_2_O_5_. The addition of the minor niobium concentration results only in a marginal decrease of the steady-state nucleation rates (Table 1), at which the maximum nucleation rate of Nb0.1 is slightly smaller than that of the undoped sample (13.52 and 16.33 1/(mm^3^ · s), respectively). However, the nucleation rates measured for 469–489 °C are very similar and the typical drop of the nucleation rates after the maximum is not observed. The maximum nucleation rate is close to T_g_ as it was also observed for the other glasses.

**Figure 1:**
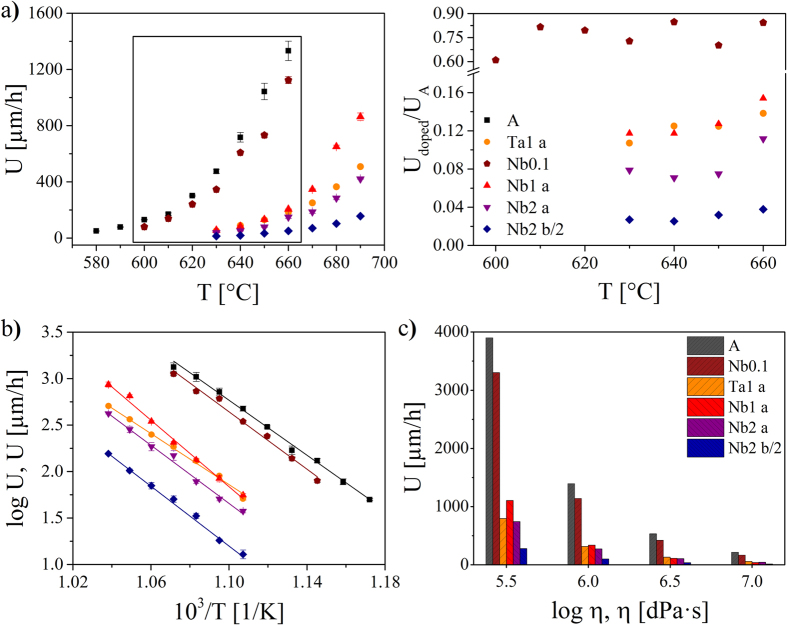
(**a**) Crystal growth velocities of the studied samples. In the case of samples Ta1, Nb1 and Nb2 different growth directions are considered. On the right hand side, the crystal growth velocities of the doped samples are related to those of sample A. In part (**b**) Arrhenius plots of the crystal growth velocity are shown while part (**c**) presents the crystal growth velocities as a function of the viscosity.

The crystal growth velocities of the studied glasses were determined by *in situ* hot stage microscopy and recently published in ref. 28. The crystal growth velocities of sample Nb0.1 are shown as a function of the temperature in Fig. 1a) (data in Table 2). It can be seen that the addition of 0.1 mol% Nb_2_O_5_ leads to a slight decrease of the crystal growth velocities which is then more pronounced with higher niobium concentrations. Since the lithium disilicate crystals possess an ellipsoidal shape and get only a minimum constriction in their center during growth, the crystal growth velocities were not determined in different growth directions as it was the case for the samples Ta1, Nb1 and Nb2^28^. Moreover, due to the comparatively high nucleation rates, a separate nucleation heat treatment step previous to the measurement of the growth velocities was not required.

### Variation in viscosity during crystallization

The crystals precipitated in samples Ta1, Nb1 and Nb2 show an approximately ellipsoidal shape with a main and a minor axis. In Fig. 1a), the crystal growth velocities are shown as a function of the temperature and growth direction (major axis a, minor axis b). It is obvious that the addition of up to 2 mol% Nb_2_O_5_ or Ta_2_O_5_ leads to a drastic decrease in the crystal growth velocities. While the crystal growth velocities of the samples Ta1 and Nb1 are almost the same in different growth directions, the crystal growth rates of sample Nb2 differ strongly. On the right hand side of Fig. 1a) the crystal growth velocities of the doped samples are related to those of samples A. With exception of sample Nb0.1, the ratio U_doped_/U_A_ increases with increasing temperature and hence lower viscosity. According to Jackson’s criteria of the interface, the crystal growth kinetics of materials with a large entropy of fusion (∆ S >  4R, R - gas constant), such as lithium disilicate, may be predicted by the screw dislocation or 2D surface nucleation model^31^,^32^. Corresponding to Nascimento *et al.* the screw dislocation model is most likely for lithium disilicate^33^. However, it is also appropriate to display the deeply undercooled region, which is solely investigated in this paper, in Arrhenius coordinates and to perform a linear approximation^33^. For this reason, the crystal growth velocities were fitted by the following equation:


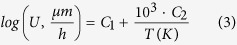


with U =  crystal growth velocity, C_1_ and C_2_ =  constants.

The corresponding parameters C_1_ and C_2_ are listed in Table 3.

As the additions of niobium and tantalum oxide to stoichiometric lithium disilicate glass lead to an increase in the viscosity, it seems likely that the drop of the crystal growth velocities is due to the higher viscosities. In order to check this assumption, the crystal growth velocities of the samples were compared at specific viscosity values. For this purpose, viscosities values obtained by beam bending and rotation viscometry were fitted by the Avramov-Milchev-equation. This enabled the calculation of viscosity values of the uncrystallized glass at temperatures where usually crystallization would occur. The fitting parameters log η _∞_, T_c_ and α of the samples are shown in Table 4. For each sample, several temperatures were calculated at which viscosity has a given value. At these temperatures, the growth rates were determined from the corresponding Arrhenius plot in Fig. 1b). In Fig. 1c), it is shown that the crystal growth velocities of the tantalum- and niobium-containing samples are much smaller also at the same viscosity than in the base glass.

#### First approximation: the viscosity depends solely on temperature

**Figure 2:**
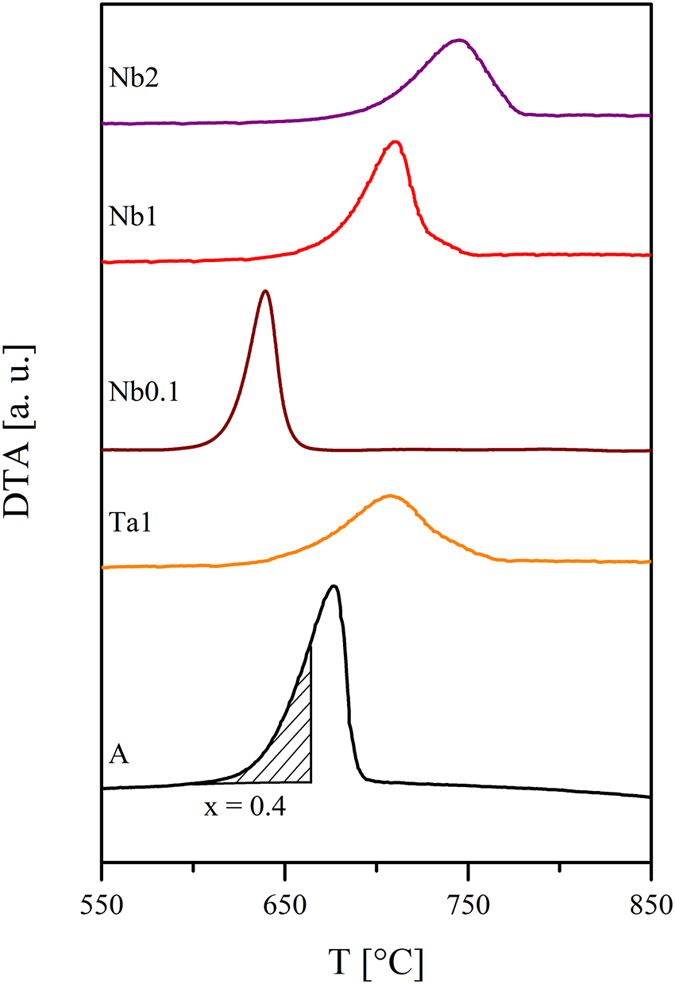
DTA profiles of the glasses. The shaded area in the crystallization peak of glass A corresponds to a transformation degree x of 0.4. The transformation degree is the ratio of the partial area at a certain temperature to the total area of the crystallization peak.

**Figure 3:**
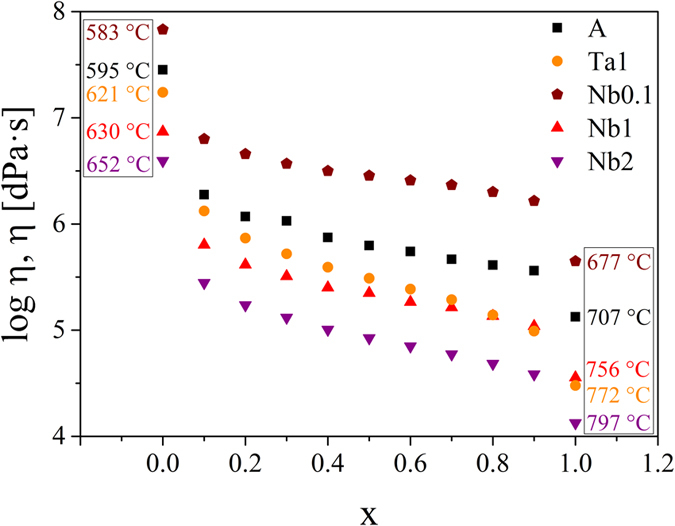
Viscosities as a function of the transformation degree x in case of the incorporation of the added oxides Nb_2_O_5_ or Ta_2_O_5_ into the lithium disilicate crystals. It should be mentioned that the behavior during a non-isothermal heat treatment is considered. For this reason the onset and endset temperatures are denoted.

In order to further investigate the effect of the viscosity on the crystal growth, the viscosities of the samples should be considered as a function of the transformation degree x, i.e. the crystallized fraction. The transformation degree is determined from a DTA profile by taking the ratio of the partial area at a certain temperature to the total area of the crystallization peak. In Fig. 2, the crystallization peaks in the DTA profiles of the studied samples as well as a schematic of the determination of the transformation degree are shown. It becomes apparent that in the case of the doped samples, the onset of the crystallization peak is shifted to higher temperatures, except for sample Nb0.1, and hence, the temperatures belonging to a particular transformation degree are totally different. In Fig. 3, the viscosities as a function of the transformation degree are illustrated. Here, the viscosities at the respective temperatures were determined from the fit with the Avramov-Milchev-equation. In this case, as a first approximation, solely the effect of changing temperatures is taken into account and the viscosity of the glass melt is considered to be not affected by changing glass compositions. Since the crystallization degree determined from DTA-scans is considered, non-isothermal behavior is illustrated. For this reason, the on- and endset temperatures of the respective crystallization peak are added for a better understanding.

While the viscosities decrease strongly at the beginning of the crystallization, the decrease weakens in case of higher transformation degrees and then is again highly pronounced. Moreover, the crystallization of the doped samples starts at a lower viscosity than in the base glass, with exception of sample Nb0.1.

#### Second approximation: the viscosity depends on the composition and changes during the course of the crystallization

**Figure 4:**
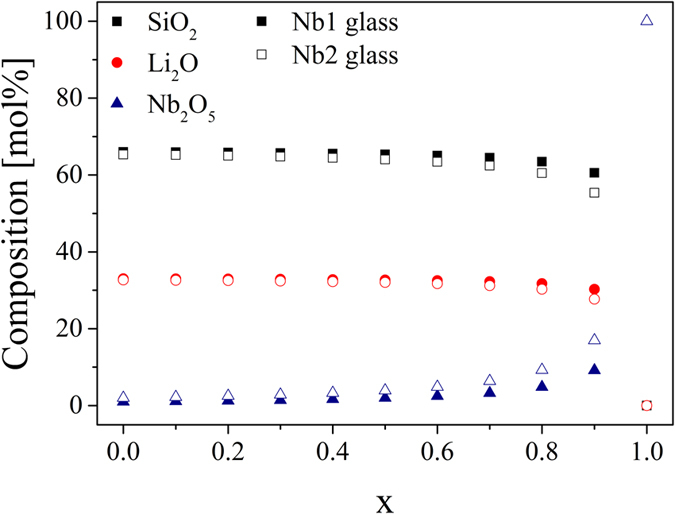
Molar composition of the residual glass matrix as a function of the transformation degree x. While the filled symbols describe the behavior of sample Nb1, the open symbols stand for the change in the composition of sample Nb2.

The results illustrated in Figs 1c and 3 are based on the assumption that the viscosity solely depends on the temperature and is not affected by changing compositions. Niobium or tantalum are not incorporated into the lithium disilicate crystals, as indicated by the X-ray diffraction patterns^28^. Hence, niobium (or tantalum) is expelled from the crystallization front leading to a continuous enrichment of the remaining glass matrix with niobium or tantalum during the course of the crystallization process. For this reason, the viscosity of the residual glass matrix has to be related to the transformation degree which requires the determination of the composition of the residual glass matrix for x >  0. The composition was calculated from the ratio of the remaining SiO_2_, Li_2_O and Nb_2_O_5_ concentrations at a certain transformation degree to their initial concentrations. The molar composition of the residual glass matrix as a function of the transformation degree is shown in Fig. 4 for the niobium-containing samples (the exact values are summarized in Table 5). The filled symbols belong to Nb1 glass and the filled symbols to Nb2 glass. Since the change in the molar composition of sample Nb0.1 is extremely small, sample Nb0.1 is not considered in Fig. 4. It is assumed that the chemical composition in the residual glassy phase is homogeneous, i.e. diffusion coefficients of the corresponding ions are much higher than the self-diffusion coefficient of the main building units, so that the profiles around the growing crystals are not formed. The change in the composition is the same in the samples Nb1 and Ta1. At the beginning of the crystallization process, a slight decrease in the molar percentage of Li_2_O and SiO_2_ is observed while the molar percentage of Nb_2_O_5_ increases. At a transformation degree of 0.5, the molar composition of sample Nb1 and hence the viscosity is the same as for the composition of sample Nb2 at x =  0. For a transformation degree, higher than 0.5, the decrease in the molar percentage of Li_2_O and SiO_2_ as well as the increase in the molar percentage of Nb_2_O_5_ is intensified. At the end, lithium disilicate crystals and a residual glassy phase with a high concentration of niobium or tantalum oxide exist.

The viscosities of the samples A, Nb1, Nb2 and Ta1 were measured using beam-bending and rotation viscometry and fitted using the Avramov-Milchev-equation at which the parameter α was kept constant (α  =  3.5). This was done due to the fact that α remained almost unchanged for small concentrations c during the fitting procedure. Moreover, log η _∞_ and T_c_ depend linearly on c:

T_c_(c) =  732 +  12c and log η _∞_(c) =  0.72 −  0.15c for the niobium-containing samples as well as T_c_(c) =  731 +  16c and log η _∞_(c) =  0.74 −  0.075c for the tantalum-containing samples. Therefore, a continuous dependency of the viscosity on both the temperature and the concentration c is given. Hence, the viscosities of the residual glass matrix were interpolated using the before mentioned equations.

**Figure 5:**
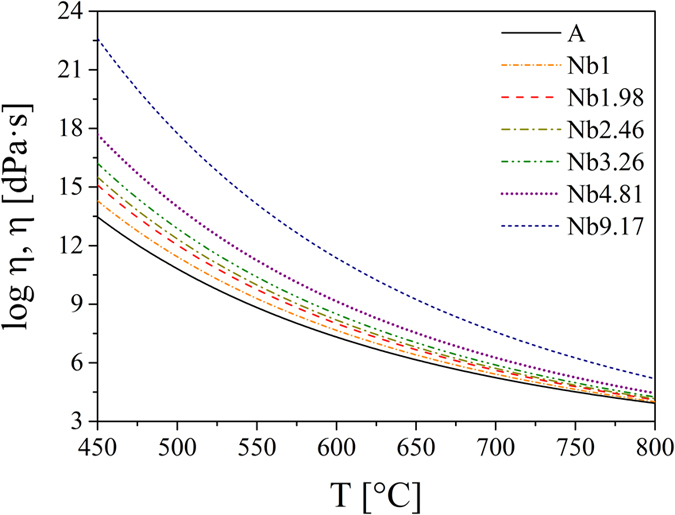
Viscosities of the base glass A and the niobium-containing glasses. The viscosities of samples A and Nb1 were measured by beam-bending and rotation viscometry. The remaining viscosities are interpolated from the measured ones.

In Fig. 5, the measured viscosities of the base glass A and the niobium-containing sample Nb1 as well as the interpolated viscosities of the residual glass matrix are shown for different Nb_2_O_5_ concentrations. The respective labels stand for the molar percentage of niobium oxide. The addition of Nb_2_O_5_ leads to a notable increase in viscosity and the effect is most pronounced at temperatures close to T_g_, i.e. in the temperature range where the nucleation rate is high, and gets smaller in the temperature range of crystal growth.

**Figure 6:**
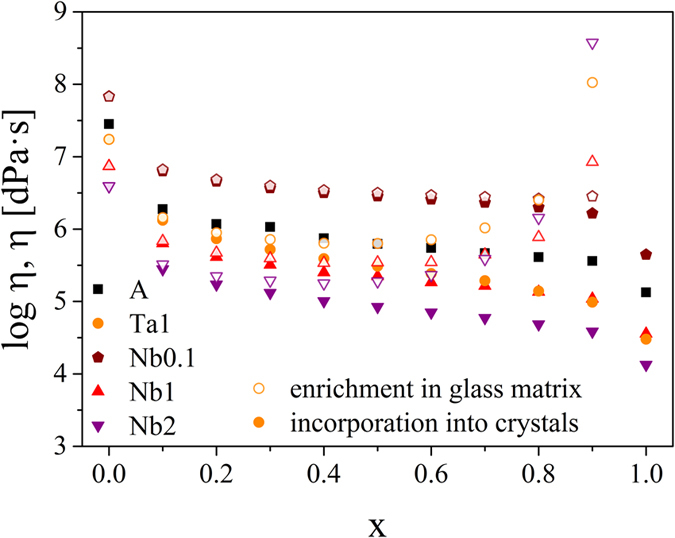
Viscosities as a function of the transformation degree x in case of the crystallization of lithium disilicate without incorporation of niobium or tantalum. It is assumed that niobium or tantalum ions are homogeneously distributed in the residual glass matrix and do not lead to an accumulation at the crystallization front.

In Fig. 6, the transformation degree is correlated with the interpolated viscosities of the residual glass matrix (open symbols, enrichment in the glass matrix). The results of Fig. 3, i.e. the case of incorporation into the crystals, are also presented for comparison (filled symbols). At the beginning of the crystallization process, the viscosity of the glass matrix initially drops strongly as it is the case in Fig. 3. However, the viscosities are higher if niobium or tantalum is enriched in the glassy matrix than for the first approximation in which the effect of changing composition was not taken into account. The difference between the two approximations gets more pronounced with an increasing degree of transformation. For a transformation degree higher than 0.4, the viscosities of the residual glass matrix increase again strongly as the molar percentage of niobium or tantalum oxide in the glass matrix increases. In the case of the small niobium oxide concentration of 0.1 mol%, the increase in viscosity only occurs at a higher transformation degree and the differences between the two approximations are not as pronounced. However, the results are based on the assumption that niobium or tantalum are homogeneously distributed in the glass matrix. Moreover, in this model an enrichment of the added oxides at the crystallization front is excluded.

**Figure 7:**
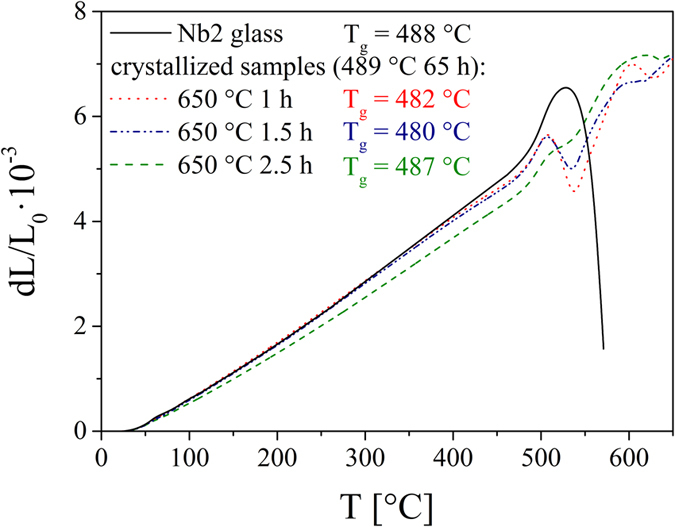
Dilatometry results of the glass Nb2 and Nb2 samples heat treated at 489 °C for 65 h and 650 °C for different times. Hence, the glass transition temperature of the residual glass matrix was determined as a function of the crystallization degree.

In the previous paragraphs, the non-isothermal crystallization process was regarded. However, based on these results it might be assumed that during an isothermal crystallization of the non-stoichiometric glasses Nb0.1, Nb1, Nb2 and Ta1, the composition of the residual glassy matrix also varies and hence, their viscosity. Therefore, pre-nucleated cylindrical samples were heat treated at a certain growth temperature for different times to obtain different crystallization degrees (Nb1 and Ta1: nucleation at 489 °C for 2 h and growth at 640 °C; Nb2: nucleation at 489 °C 65 h and growth at 650 °C) and the glass transition temperature T_g_ of the residual glass matrix was determined by dilatometry. In Fig. 7, the results from dilatometry are shown for crystallized Nb2 samples along with the corresponding glass sample. While the glass sample has a T_g_ at 488 °C, the T_g_ initially decreases with increasing holding time at the growth temperature, i.e. increasing crystallization degree, and then again increases. The differences in T_g_ are extremely small and within the limits of error (temperature and evaluation error). However, the same behavior was also observed for the other glasses and it is in agreement with the results of Fig. 6. In addition, in Fig. 7 it can be seen that the well pronounced softening point of the glass sample does not occur in the crystallized samples. Instead, only a slight softening point followed by a further increase in dilatation and a second softening are observed probably due to the high crystallization degree which impedes softening.

### Activation energy for crystallization

**Figure 8:**
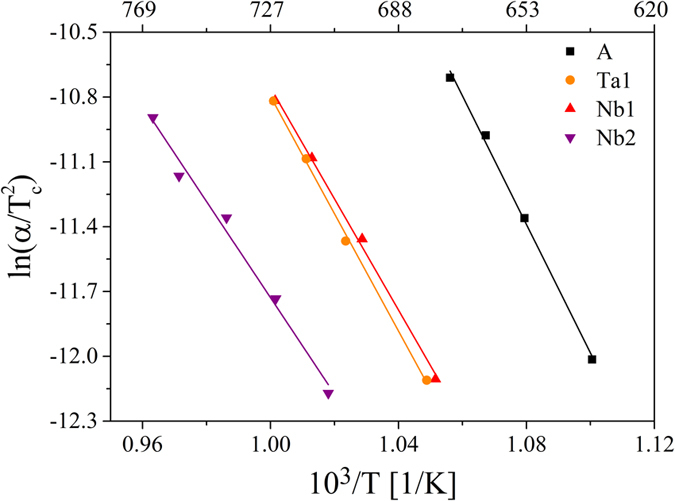
Kissinger plots of the samples A, Ta1, Nb1 and Nb2. The activation energy for crystallization was determined from the slope of the regression line.

The activation energy for crystallization may be determined from the Kissinger^34^ or Ozawa^35^ equations. In these relations, the shift of the crystallization temperature to higher values and the increasing area under the crystallization peak with increasing heating rate are used. Both equations are valid if surface crystallization is the predominant crystallization mechanism or for n =  m (n - Avrami parameter which corresponds to the nucleation and growth mechanism, m - dimensionality of crystal growth)^36^. The latter is applicable if the number of nuclei is independent of the applied heating rate, i.e. crystallization occurs from the same number of nuclei at different heating rates^37^. The condition n =  m is fulfilled since the number of nuclei, counted after annealing samples to the development temperature with different heating rates in the heating stage of the laser scanning microscope, was approximately equal. The obtained Kissinger plots are shown in Fig. 8 and the calculated activation energies for crystallization are summarized in Table 6 along with the activation enthalpies for crystal growth E_a_ calculated from Arrhenius plots of the crystal growth velocities. It should be mentioned that the activation energy computed using the Kissinger method describes the overall crystallization process including nucleation and crystal growth. In Table 6, it can be seen that the activation energies E_Kissinger_ of the doped samples are lower than that of the base glass and that with increasing niobium concentration, the activation energy also decreases. By contrast, the activation enthalpy for crystal growth of the sample Ta1 is slightly smaller and those of the niobium-containing samples are slightly to considerably higher than that of sample A^28^.

## Discussion

In the past decades, stoichiometric lithium disilicate glass has been the subject of numerous investigations concerning nucleation and crystal growth kinetics^3^,^5^,^6^,^38^,^39^, especially regarding the applicability of the classical nucleation theory^4^,^40^,^41^,^42^. Moreover, the effect of small amounts of additives which promote nucleation (e.g. water^43^, platinum^44^,^45^,^46^,^47^ or P_2_O_5_^48^,^49^,^50^) on the nucleation rates as well as on the crystal growth velocities was studied. In the case of the addition of water, the effect was reported to be related to a decrease of the kinetic barrier for nucleation due to lower viscosities, rather than changing the thermodynamic barrier of nucleation, i.e. decreasing the surface energy^43^. Krüger *et al.* added up to 12 wt% ZrO_2_ to a SiO_2_/Li_2_O (− K_2_O/Al_2_O_3_/P_2_O_5_) glass and detected that an increasing zirconia concentration leads to an increase in the nucleation rates by about two orders of magnitude^51^. Here, it was assumed that ZrO_2_ likely affects the thermodynamic barrier of nucleation (see Eq. 1)^51^. Matusita and Tashiro measured nucleation rates in alkali disilicate glasses (Li, Na and K) and observed the highest nucleation rates in Li_2_O · 2SiO_2_ glass^52^. This effect was attributed to the great free energy difference in this system, i.e. to the thermodynamic driving force and not related to the viscosity or the interfacial energy^52^.

However, the effect of nucleation inhibitors leading to decreasing nucleation rates and crystal growth velocities as well as to increasing induction times for steady-state nucleation has only been scarcely investigated in the past. Matusita and Tashiro^53^ as well as Schlesinger and Lynch^54^ have already analyzed the effect of small concentrations of oxides on the nucleation kinetics of lithium disilicate glass and in these publications the nucleation inhibition was attributed to higher viscosities of the glasses. However, a recent paper showed that the addition of Al_2_O_3_, La_2_O_3_ and TiO_2_ to stoichiometric lithium disilicate glass leads to a drastically decrease in the nucleation rates at certain viscosity values^26^. Moreover, it was observed that the effects are much more pronounced than the increase in viscosity would provoke^26^. The addition of ZrO_2_ concentrations to Li_2_O · 2SiO_2_-glass leads to remarkably smaller nucleation rates and interestingly for zirconia concentrations > 2 mol%, volume nucleation was not observed and instead only surface crystallization was detected^27^. The absence of volume nucleation was correlated with the reduced glass transition temperature and is explained by the higher work for critical cluster formation in the bulk than at the surface at temperatures near T_g_^55^,^56^,^57^. Nucleation inhibition occurs also in Nb_2_O_5_- or Ta_2_O_5_-doped lithium disilicate glasses which was related to a decreased structural similarity between glass and crystal as it is assumed that the added oxides are incorporated as distorted MO_6_-octahedra (M =  Nb, Ta) in the glass matrix^28^. Furthermore, in all cases a higher stability against crystallization is observed which is associated with slower crystallization^28^. Moreover, the additives lead to a remarkable decrease of the crystal growth velocities^26^,^27^,^28^. Recently, in lithium disilicate glasses doped with CeO_2_, cerium accumulations between the lamellae of the lithium disilicate crystals and at the crystallization front were detected using fluorescence and scanning electron microscopy resulting in decreased crystal growth velocities^58^.

According to the Classical Nucleation Theory (CNT), the steady-state volume nucleation rate I_st_ can be described by the following equation^59^


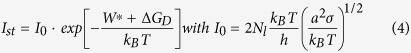


where I_0_ is a preexponential term consisting of the number of structural units N_l_ per unit volume of the melt with a mean size a, the Boltzmann constant k_B_, the Planck constant h and the surface energy of the nucleus/melt interface per unit area σ. While W^*^ is the thermodynamic barrier for nucleation, i.e. the work of critical cluster formation, ∆ G_D_ describes the kinetic barrier for nucleation, i.e. the activation energy for the transport of a structural unit across the nucleus/melt interface. Assuming a spherical nucleus the thermodynamic barrier for nucleation W^*^ can be described by


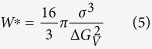


The thermodynamic driving force for crystallization ∆ G_V_ which is the difference between the free energies of the glass and the crystalline phase per unit volume^60^ can be estimated from the melting enthalpy per unit volume of the crystal ∆ H_m_ at the melting temperature T_m_ assuming that ∆ C_p_ =  0 (difference of the molar heat capacities of melt and crystal at constant pressure)^39^:


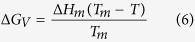


The kinetic barrier for nucleation ∆ G_D_ is often described by an effective diffusion coefficient D (ref. 50) which is given by:


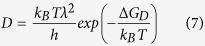


Here, λ describes a jump distance of the order of atomic dimensions. Moreover, D can be related to the viscosity η by the following equation^50^:


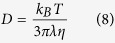


The time required to reach steady-state nucleation, i.e. the time-lag for nucleation τ , is given by equation (9) if the initial concentration of critical and sub-critical nuclei is negligible^57^:


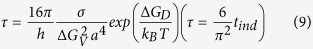


**Figure 9:**
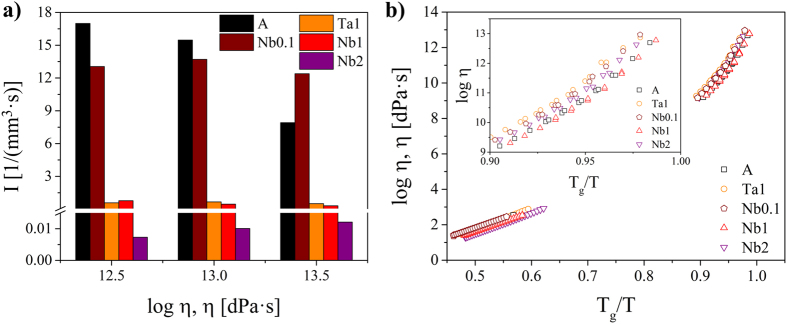
(**a**) Steady-state nucleation rates of the studied samples for different viscosities (12.5, 13 and 13.5 dPa · s) and (**b**) viscosities as a function of the ratio T_g_/T (T_g_ determined by DTA).

According to equation (4), small crystal nucleation rates might be due to an increase in both the thermodynamic (W^*^) and the kinetic barrier for nucleation (∆ G_D_). In Fig. 9a), the steady-state nucleation rates of the base glass A are compared to those of Nb0.1 and Ta1 at specific viscosity values (12.5, 13 and 13.5 dPa · s). It becomes apparent that the nucleation rates of Ta1 are up to two orders of magnitude lower at the same viscosity values while those of Nb0.1 are in a similar range as the base glass A. Moreover, it can be seen that the nucleation rates of Ta1 and Nb1 are approximately the same while those of Nb2 are one order of magnitude lower. In part b), the beam bending and rotation viscosities are presented as a function of T_g_/T (T_g_ determined by DTA^26^,^28^) and it can be seen that the viscosities of the glasses match very well. In the inset, showing T_g_/T-values between 0.9 and 1.0 (i.e. the range of nucleation above T_g_), it is visible that there are only minimal deviations between the glasses which, however, are not related to the nucleation rates and in addition the slight viscosity differences might not provoke such high discrepancies in the nucleation rates. Based on these facts, the viscosity has to be excluded as a key factor for nucleation inhibition.

The formation of critical nuclei and the subsequent crystal growth is strongly affected by the attachment rate, also called impingement rate, of building units at the surface of the nuclei and the growing crystals, respectively. According to refs 61 and 62, the steady-state nucleation rate and the nucleation time-lag are also defined as (see for comparison equations (4) and (9))


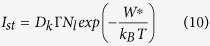



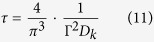


While D_k_ is the molecular flux to the critical nucleus^61^, Γ describes the Zeldovich factor which converts an equilibrium expression into a steady-state expression^63^. The flux D_k_ is given by the product of the surface area of the nucleus A_k_, the impingement rate per unit area Z of the structural units to the nucleus and a numerical factor ζ which characterizes the steric hindrance at the nucleus interface during the incorporation of the building units^61^. According to ref. 61, for undercooled melts the impingement rate Z is


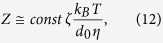


where d_0_ is the mean atomic distance. Since the viscosity as key factor for nucleation inhibition was excluded in the last paragraph, the incorporation of the building units seems to be hindered by the layer of the added oxides at the interface which is indicated by the decrease in the steady-state nucleation rates with increasing dopant concentration. Furthermore, it was shown that a minor concentration of 0.1 mol% Nb_2_O_5_ may slightly affect the nucleation rates which is contradictory to a study of James *et al.*^64^ who investigated the effect of different crucible and stirrer materials as well as the purity of the raw materials on crystal nucleation kinetics in lithium disilicate glass. They observed that the quantities of impurities present in ordinary batch materials and different melt conditions do not remarkably affect the nucleation rates. However, the present results indicate that the effect of impurities on nucleation and crystal growth kinetics is not negligible.

**Figure 10:**
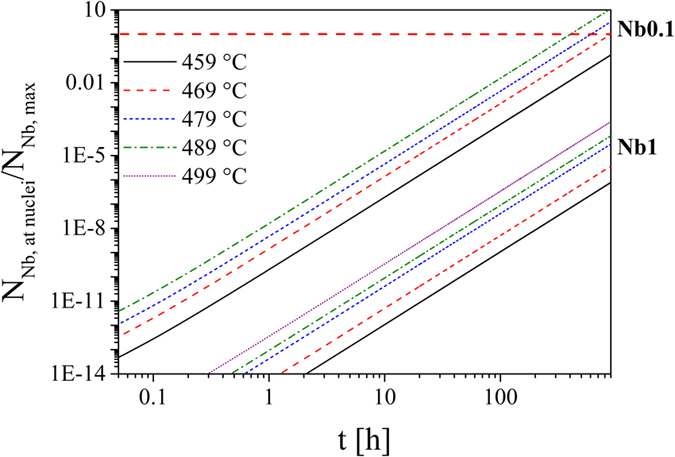
Ratio of the number of Nb-particles required for a complete coverage of the nuclei (N_Nb,at nuclei_) and the number of available Nb-particles in the considered volume (N_Nb,max_) versus holding time at different nucleation temperatures for Nb0.1 and Nb1.

Based on the Nb_2_O_5_ concentration in the glasses, the glass density and the nucleation rates, it is possible to estimate whether the niobium concentration is sufficient for a complete coverage of the nuclei during the nucleation process. In this calculation, the lithium disilicate nuclei are assumed to be spherical. Moreover, since the exact attachment process and hence the packing factor is unknown a hexagonal packing of the Nb-O-groups (factor 0.907) at the surface of the nuclei is supposed in the following. In these calculations a nuclei size distribution at the respective nucleation temperature is considered. In Fig. 10, the ratio of the number of Nb-particles which is necessary for a complete coverage of the nuclei (N_Nb,at nuclei_) and the number of available Nb-particles in the considered volume (N_Nb,max_) is shown as a function of the holding time at different nucleation temperatures. The crystal growth velocities at the nucleation temperatures are extrapolated using the regression parameters stated in Table 3. In the case of samples Nb1 and Nb2, the growth direction a is regarded. For a better understanding, it should be mentioned that if the ratio N_Nb,at nuclei_/N_Nb,max_ is higher than 1, the number of Nb-particles in this volume is too low for a complete coverage of the nuclei (highlighted by a red line). It is visible that the holding time for reaching the equilibrium between required and available particles decreases with increasing nucleation temperature. However, in the case of Nb1 and Nb2 (not shown in this graph) only a fraction of the available Nb-particles can be attached at the nuclei interface, even after 900 h, due to the low nucleation rates and crystal growth velocities. Since the nucleation rates of Nb1 and Nb2 differ by one order of magnitude, the ratio N_Nb,at nuclei_/N_Nb,max_ is also one order of magnitude lower in sample Nb2. In sample Nb2, the higher niobium concentration is not sufficient to compensate the lower number of nuclei. In comparison to the hexagonal packing which is described before, a quadratic packing of the Nb-O-groups with a packing factor of 0.785 would result in a higher N_Nb,at nuclei_ at the respective nucleation temperature and time and hence the ratio N_Nb,at nuclei_/N_Nb,max_ would be increased. As a result, the nuclei surface would be covered earlier by the Nb-O-diffusion barrier.

**Figure 11:**
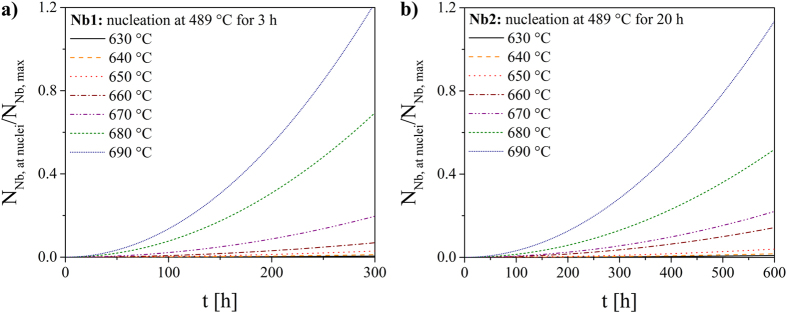
Ratio N_Nb,at nuclei_/N_Nb,max_ as a function of the holding time at various growth temperatures. The calculations are based on the number of nuclei formed during a nucleation treatment at 489 °C for 3 h (Nb1) and 20 h (Nb2).

In Fig. 11, the number of niobium particles during isothermal crystal growth at 630–690 °C is considered. The number of existing nuclei was calculated for the applied nucleation treatments at 489 °C for 3 h (Nb1) and 20 h (Nb2) and the crystal growth velocities published in ref. 28 were used. In the case of sample Nb1 and Nb2 extremely long holding times at the growth temperatures are necessary for reaching the point where the number of Nb-particles is too low for covering the nuclei. However, the calculated times are unrealistic as the samples are completely crystallized after a few hours.

Based on these calculations, it can be concluded that the niobium concentrations, especially in the samples Nb1 and Nb2, are sufficient to provoke a niobium oxide layer at the interface and hence may decrease the impingement rate.

At a first glance, the nucleation inhibition might also be a result of a higher thermodynamic barrier of nucleation which, in principle might be caused by a higher specific interface energy of the nucleus/melt interface σ , or a smaller thermodynamic driving force for crystallization ∆ G_V_. The added oxide might only affect the specific interface energy if it occurs at the interface crystal/residual glass matrix. This, however, should only be the case if it is thermodynamically advantageous. As a result, the specific interface energy would be decreased. Since the added oxides are adsorbed at the interface, it is likely that the surface energy of the nucleus/melt interface is decreased by the additives. Otherwise, the layer at the crystal interface would not be formed as it is thermodynamically disadvantageous. Unfortunately, due to the lack of a reliable method, the interfacial energy is not experimentally accessible. Therefore, experimental nucleation data are fitted to the CNT. However, according to CNT, it is supposed that the thermodynamic properties of the nuclei are similar to the corresponding macroscopic phase (capillarity approximation). For this reason, the nucleus/melt surface energy is treated as a planar interface of the macro-phase^60^. Moreover, besides the size dependency, the temperature dependency of the surface energy is mostly not considered^60^. Although the surface energy of glass melts shows only a slight temperature dependency (usually ~1%/100 K), this might also affect the nucleation process.

According to Eq. 2, the thermodynamic barrier for nucleation W^*^ is also increased by a diminished thermodynamic driving force for crystallization ∆ G_V_. According to refs 65 and 66, a deviation of the nucleus composition from that of the stoichiometric glass or the initial glass composition generally results in a decreased ∆ G_V_. Hence, the addition of niobium or tantalum oxide or any other component to the glass composition should alone be sufficient to decrease the thermodynamic driving force and hence to decrease the nucleation rate. Small concentrations of additives, however, should not have a large effect.

Furthermore, the formation of stoichiometric crystals may proceed through the nucleation of solid solutions^55^,^65^. Based on the aforementioned publications, the nucleation of lithium disilicate solid solutions may not be completely excluded in the studied glasses. Moreover, it is also possible that trace quantities of niobium or tantalum are incorporated into the lithium disilicate crystals, without detecting them with the applied methods due to the detection limits. The thermodynamic driving force can be further decreased by the occurrence of metastable phases which may also result in a deviating nucleus composition^59^. In the studied lithium disilicate system, the precipitation of the metastable lithium metasilicate in Li_2_O · 2SiO_2_ glasses has been a heavily discussed issue in the past decades (e.g. refs 2 and 67, 68, 69, 70). However, the precipitation of lithium metasilicate (in the early stages of crystallization) was not observed in our glass compositions. Elastic stresses evolving during the course of the crystallization due to a difference between the densities of the glass and the crystal might lead to a decrease in ∆ G_V_, especially for temperatures lower than the so-called decoupling temperature (T_d_ ~ 1.2T_g_)^59^. It was shown for lithium disilicate glass that the steady-state nucleation rates may be decreased by up to two orders of magnitude due to elastic stresses^71^. However, the comparison of the densities of the studied glasses with the crystal density (ρ _crystal_ =  2.45 g/cm^3^, ref. 72), assuming the precipitation of stoichiometric lithium disilicate crystals, shows that the difference in the densities is highest for sample A and decreases if Nb_2_O_5_ or Ta_2_O_5_ are added. For this reason, the effect of elastic stresses on the thermodynamic driving force cannot be responsible for the observed effect.

If a breakdown of the Stokes-Einstein-equation at temperatures lower than the decoupling temperature (T_d_ ~ 1.2T_g_) is neglected and the kinetic barrier for nucleation is connected with the glass viscosity, equation (4) can be transformed to^30^,^57^:


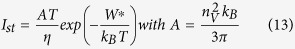


where n_v_ is the number of formula units per unit volume of the glass. Hence, the thermodynamic barrier for nucleation can be calculated as follows:


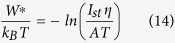


**Figure 12:**
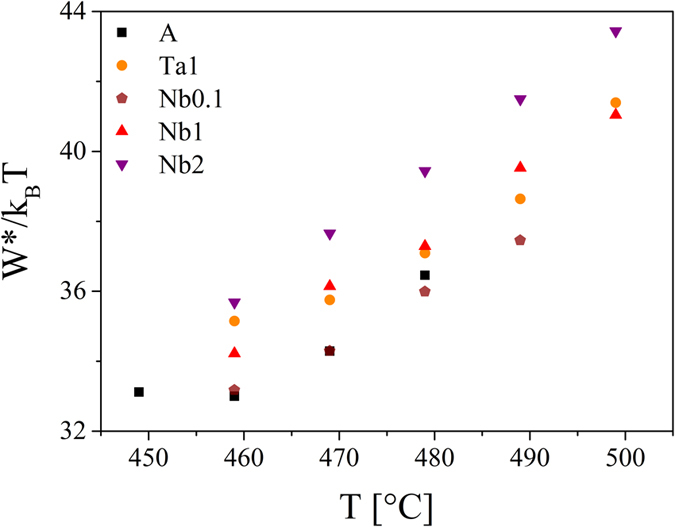
Thermodynamic barrier for nucleation as a function of the temperature.

Based on the fact that neither niobium nor tantalum is incorporated into the lithium disilicate crystals, the preexponential factor A is the same for all studied glasses. According to ref. 4, n_v_ is typically 10^29^ m^−3^. Fig. 12 shows W*/k_B_T as a function of the temperature for the different glasses. It becomes apparent that the thermodynamic barrier of the doped samples is considerably higher than that of the base glass. Only sample Nb0.1 which exhibits nucleation rates in the same order of magnitude compared with sample A has similar W*/k_B_T-values. The small increase in the thermodynamic barrier for nucleation of sample A at temperatures below 460 °C was also observed in refs 30 and 57.

As mentioned in the previous paragraphs, the thermodynamic barrier for nucleation is determined by the thermodynamic driving force ∆ G_V_ and the interfacial energy crystal/glassy matrix σ . Using the melting enthalpy per unit volume ∆ H_m_ and the melting temperatures T_m_, ∆ G_V_ can be computed using the Turnbull approximation in equation (6). Since ∆ H_m_ is proportional to the melting peak area, it can be roughly estimated by DSC. The absolute measurement error of T_m_ is approximately ± 5 K. Furthermore, ∆ H_m_ of sample A is in a good agreement with the literature data (57.3 kJ/mol ≡ 9.36 · 10^8^ J/m^3^ (ref.^ 42^) and 57.4 kJ/mol ≡ 9.38 · 10^8^ J/m^3^ (used in ref. 4, average of the values given in refs 73 and 74)). As shown in Table 7, the values of ∆ H_m_ for the doped samples are clearly smaller than for sample A.

**Figure 13:**
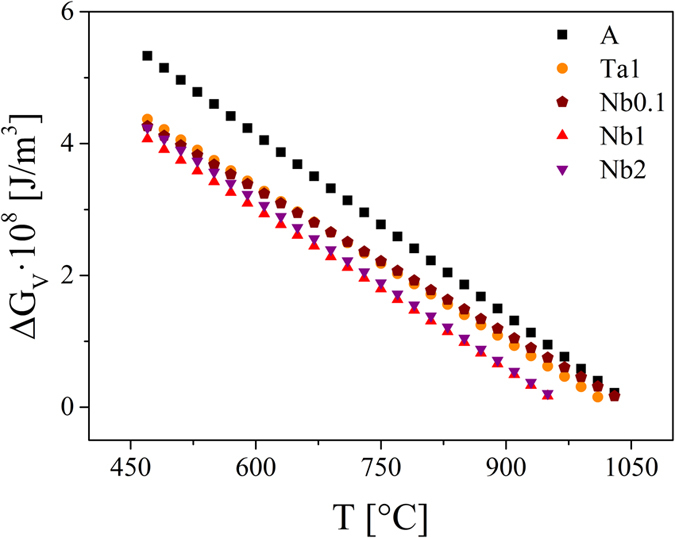
Thermodynamic driving force per unit volume of the lithium disilicate crystallization as a function of the temperature.

As can be seen in Fig. 13, the thermodynamic driving force of the base glass is the highest, while the samples Nb1 and Nb2 show the lowest ∆ G_V_. It should be mentioned that the Turnbull expression gives an upper bound for ∆ G_V_(T) and is only valid if ∆ C_p_ =  0.

The interfacial energy σ can be calculated using the semi-empirical Turnbull-Skapski-equation^59^. In this equation, it is assumed that σ _∞_ does not depend on the temperature and the size of the nucleus.


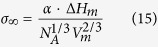


While α is an empirical dimensionless coefficient (for lithium disilicate glass α  =  0.44, ref. 30), N_A_ is Avogadro’s number. In the literature, σ _∞_ of Li_2_O · 2SiO_2_ was calculated from the slope of the plot ln(Iη /T) vs. 1/(Δ G^2^T) yielding the following results: 0.196 J/m^2^ (Matusita & Tashiro^52^), 0.207 J/m^2^ (Weinberg *et al.*^42^) and 0.198–0.209 J/m^2^ (Zanotto & James^4^). In Table 7, it is visible that the calculation of σ _∞_ of the undoped glass using equation (15) results in the slightly higher value 0.245. Moreover, σ _∞_ is slightly decreased by the addition of Nb_2_O_5_ or Ta_2_O_5_ at which the values are all within the limits of error.

Hence, the assumptions that the thermodynamic driving force as well as the interfacial energy are diminished could be confirmed by the calculations. Although σ is included in equation (5) with the third power, the decrease of ∆ G_V_ seems to be the determining factor for the increase of W*/k_B_T (Fig. 12).

As shown in Fig. 6, during the course of the crystallization, the composition of the residual glass matrix changes in the non-stoichiometric samples leading to an increase of the viscosity. For simplification, the model is based on the assumption that niobium or tantalum oxide is homogeneously distributed in the glassy matrix. However, as mentioned previously, the added oxides are adsorbed at the crystal/glass matrix interface forming a diffusion barrier. The self-limited crystal growth due to a diffusion zone has been already reported for e.g. oxyfluoride glasses^7^,^16^, apatite^12^ and quartz from the systems MgO-Al_2_O_3_-SiO_2_^10^,^75^ or Li_2_O-Al_2_O_3_-SiO_2_^9^. Here, the composition in the vicinity of the crystals changes leading to a drastic change of the glass structure^15^. If an enrichment of network former occurs near the nuclei, crystal growth will decelerate with time. The crystal growth velocities remarkably decrease by the added oxides and in addition with increasing niobium concentration (Fig. 1). Hence, these effects may be likely related to an enrichment of the additives at the crystallization front as explained in the previous paragraph. This leads to a decrease of the interfacial energy crystal/glassy phase which alone should not decrease, but increase the nucleation rate. This effect, however, is overcompensated by the decrease in the thermodynamic driving force and supposedly most important by an increase in the kinetic barrier, ∆ G_D_, and a lower impingement rate Z, due to the blocking effect of the niobium or tantalum enriched layer around the subcritical nuclei.

In summary, this leads to deceleration of nucleation and crystal growth and to an increase in the induction period.

## Conclusions

Steady-state nucleation rates and crystal growth velocities of stoichiometric lithium disilicate glass significantly decrease by the addition of small niobium or tantalum oxide concentrations. Even a minor concentration of 0.1 mol% Nb_2_O_5_ provokes a slight inhibition of the nucleation and growth kinetics. In the course of non-isothermal crystallization of the glasses, the viscosity of the residual glass matrix initially decreases and then remarkably increases with increasing crystallization degree hindering further crystal growth. This is based on the assumption that the niobium and tantalum ions are not incorporated into the lithium disilicate crystals but homogeneously distributed in the glass matrix. In the case of an isothermal heat treatment of the samples, a similar viscosity trend is observed. However, it was shown that the added oxides are accumulated at the crystallization front resulting in a lower interfacial surface energy. Since a lower interfacial surface energy would alone provoke higher nucleation rates, this has to be compensated by a decreased thermodynamic driving force. The viscosity has to be excluded as key factor as the nucleation rates of the doped samples are even drastically lower at the same viscosity values. However, due to the accumulation of the additives at the crystallization front, the interface of the nuclei is blocked and the attachment of further building units is decelerated. Hence, the impingement rate is lowered with increasing additive concentration.

In summary, the addition of small amounts of the studied oxides enables the control of the crystallization behavior of the glasses in a wide range. This is solely limited by a maximum additive concentration at which the ions are incorporated into the crystals.

## Additional Information

**How to cite this article**: Thieme, K. *et al.* The mechanism of deceleration of nucleation and crystal growth by the small addition of transition metals to lithium disilicate glasses. *Sci. Rep.* **6**, 25451; doi: 10.1038/srep25451 (2016).

## Figures and Tables

**Table 1:** Steady-state nucleation rates I and corresponding induction times t_ind_ of the glass Nb0.1 compared with the base glass A as a function of the temperature.

T [°C]	I [1/(mm^3^ · s)]		t_ind_ [min]	
	A^a^	Nb0.1	A^a^	Nb0.1
449	1.22	b	167	b
459	10.02	6.99	104	58
469	16.33	13.51	25	14
479	9.25	12.31	–	–
489	b	12.17	b	–

I and t_ind_ were determined by a regression analysis (least squares method) with a correlation coefficient R^2^ >  0.96. In the case of the nucleation rates, there is an uncertainty of the last digit.

^a^Values taken from ref. 26.

^b^Not measured.

**Table 2:** Crystal growth velocities of sample Nb0.1.

T [°C]	U [μm/h]	
	A^a^	Nb0.1
580	50 ± 1	–
590	78 ± 3	–
600	131 ± 3	80 ± 2
610	169 ± 9	138 ± 3
620	302 ± 4	240 ± 2
630	475 ± 15	346 ± 4
640	717 ± 34	608 ± 3
650	1,043 ± 58	732 ± 15
660	1,332 ± 69	1,124 ± 25
E_a_ [kJ/mol]	283 ± 4	296 ± 13

^a^Values taken from ref. 26.

**Table 3:** Regression parameters determined by fitting the crystal growth velocities with the regression equation 
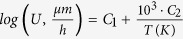
 .

Sample	Direction	C_1_	C_2_ [K]
A		19.1 ± 0.2	− 14.8 ± 0.2
Ta1	a	16.9 ± 0.4	− 13.7 ± 0.4
Nb0.1		19.6 ± 0.8	− 15.4 ± 0.7
Nb1	a	21.5 ± 0.9	− 17.9 ± 0.8
Nb2	a	18.9 ± 0.3	− 15.6 ± 0.3
	b/2	18.9 ± 0.3	− 16.1 ± 0.3

**Table 4:** Parameters log η_∞_ and T_c_ obtained by fitting the viscosity graphs with the Avramov-Milchev-equation with α = 3.5.

Sample	log η_∞_ [dPa·s]	T_c_ [K]	α
A	0.73 ± 0.01	731.0 ± 0.1	3.5 ± 0
Ta1	0.68 ± 0.00	746.8 ± 0.1	3.5 ± 0
Nb0.1	0.69 ± 0.00	732.8 ± 0.1	3.5 ± 0
Nb1	0.56 ± 0.00	743.9 ± 0.1	3.5 ± 0
Nb2	0.41 ± 0.00	755.1 ± 0.1	3.5 ± 0

**Table 5:** Calculated molar composition of the residual glass matrix as a function of the transformation degree x.

x	Nb0.1 glass			Nb1/Ta1 glass			Nb2 glass		
	Li_2_O	SiO_2_	Nb_2_O_5_	Li_2_O	SiO_2_	Nb_2_O_5_/Ta_2_O_5_	Li_2_O	SiO_2_	Nb_2_O_5_
0	33.3	66.6	0.10	33	66	1	32.67	65.33	2
0.1	33.3	66.59	0.11	32.96	65.93	1.11	32.59	65.19	2.22
0.2	33.29	66.58	0.12	32.92	65.84	1.25	32.50	65.01	2.49
0.3	33.29	66.57	0.14	32.86	65.72	1.42	32.39	64.78	2.83
0.4	33.28	66.56	0.17	32.78	65.56	1.66	32.24	64.47	3.29
0.5	33.27	66.53	0.20	32.67	65.35	1.98	32.03	64.05	3.92
0.6	33.25	66.50	0.25	32.51	65.02	2.46	31.72	63.43	4.85
0.7	33.22	66.44	0.33	32.25	64.50	3.26	31.21	62.42	6.37
0.8	33.17	66.33	0.50	31.73	63.46	4.81	30.25	60.49	9.26
0.9	33.00	66.01	0.99	30.28	60.55	9.17	27.68	55.37	16.95
1.0	0	0	100	0	0	100	0	0	100

**Table 6:** Activation energies for crystallization determined by the Kissinger method.

Sample	E_Kissinger_ [kJ/mol]	Preexponential factor	Direction	E_a_ [kJ/mol]^a^
A	247 ± 9	20.73		283 ± 4
Ta1	226 ± 6	16.35	a	263 ± 8
			b	269 ± 8
Nb1	214 ± 8	14.96	a	342 ± 16
			b	348 ± 15
Nb2	185 ± 12	10.54	a	299 ± 5
			b/2	308 ± 6

The activation enthalpies for crystal growth E_a_ obtained from Arrhenius plots of the crystal growth velocities are shown for comparison.

^a^Values taken from refs 26 and 28.

**Table 7:** Melting temperatures T_m_, melting enthalpies ∆H_m_ and surface energies σ_∞_ of the studied samples.

Sample	T_m_ [°C]	∆H_m_ [J/m^3^]	σ_∞_ [J/m^2^]
A	1034	1.19 · 10^9^	0.245
Ta1	1010	1.00 · 10^9^	0.206
Nb0.1	1033	9.56 · 10^8^	0.196
Nb1	951	9.95 · 10^8^	0.206
Nb2	955	1.03 · 10^9^	0.212

The measurement error of T_m_ is approximately ± 5 K. The error in the estimation of σ _∞_ is about 0.02 J/m^2^.
